# Understanding the Determinants of Sweet Liking in the African and East Asian Ancestry Groups in the U.S. – A Study Protocol

**DOI:** 10.21203/rs.3.rs-3644422/v1

**Published:** 2023-11-28

**Authors:** May M Cheung, Patrice A Hubert, Danielle R Reed, Enrique R Pouget, Xinyin Jiang, Liang-Dar Hwang

**Affiliations:** City University of New York, Brooklyn College; Monell Chemical Senses Center; Monell Chemical Senses Center; City University of New York, Brooklyn College; City University of New York, Brooklyn College; The University of Queensland

## Abstract

**Background:**

The liking for sweet taste is a powerful driver for consuming added sugars, and therefore, understanding how sweet liking is formed is a critical step in devising strategies to lower added sugars consumption. However, current research on the influence of genetic and environmental factors on sweet liking is mostly based on research conducted with individuals of European ancestry. Whether these results can be generalized to people of other ancestry groups warrants investigation.

**Methods:**

We will determine the differences in allele frequencies in sweet-related genetic variants and their effects on sweet liking in 426 adults of either African or East Asian ancestry, who have the highest and lowest average added sugars intake, respectively, among ancestry groups in the U.S. We will collect information on participants’ sweet-liking phenotype, added sugars intake (sweetness exposure), anthropometric measures, place-of-birth, and for immigrants, duration of time living in the U.S. and age when immigrated. Ancestry-specific polygenic scores of sweet liking will be computed based on the effect sizes of the sweet-related genetic variants on the sweet-liking phenotype for each ancestry group. The predictive validity of the polygenic scores will be tested using individuals of African and East Asian ancestry from the UK Biobank. We will also compare sweet liking between U.S.-born individuals and immigrants within each ancestry group to test whether differences in environmental sweetness exposure during childhood affect sweet liking in adulthood.

**Discussion:**

Expanding genetic research on taste to individuals from ancestry groups traditionally underrepresented in such research is consistent with equity goals in sensory and nutrition science. Findings from this study will help in the development of a more personalized nutrition approach for diverse populations.

**Trial registration:**

This protocol has been preregistered with the Center for Open Science (https://doi.org/10.17605/OSF.IO/WPR9E) and is approved by the City University of New York Human Research Protection Program (IRB#: 2023–0064-Brooklyn).

## Background

The pleasure derived from sweet taste is a major reason for consuming too much added sugars^1–4^. Because of the potential negative health outcomes of the overconsumption of added sugars, leading health agencies recommend reducing its intake to improve health^5^. For example, the *American Heart Association* recommends limiting the intake of added sugars to no more than 9 teaspoons per day for men and 6 teaspoons per day for women and children^5^, in contrast to the average of 17 teaspoons per day on average that Americans consume^6^. However, reducing added sugars consumption is not easy for many people, and it can be even more challenging for people who like sweeter foods and beverages (a.k.a., having a “sweet tooth”). To develop successful personalized nutrition strategies to reduce the intake of added sugars, there is a need to better understand the factors that contribute to individual variations in sweet liking.

A person’s liking for sweetness has a genetic origin but may also be influenced by environmental factors^7–12^. Human twin studies have found that individual differences in sweet taste liking are partly inherited, with the estimated heritability ranging from 27–41% in a U.S. cohort^13^ and a Finnish cohort^9^, respectively. Genome-wide association studies (GWAS) have further identified single-nucleotide polymorphisms (SNPs) within several genes to be associated with sweet liking and sweet food intake in a study of European ancestry using data from the U.S., Australia, and UK Biobank^7,14^. On the other hand, studies investigating the effects of environmental influences, such as repeated exposure to sweet tastes, have yielded equivocal results^10,12,15–17^. While one study found that repeated exposure to sweet taste in infants less than 12 months old did not lead to increased sweet liking^12^, another clinical trial reported that adults who reduced their sugar-sweetened beverage consumption showed a decreased preference for sweetness after 12 months^10^. Overall, both genetics and environmental factors should be considered to better understand how individual sweet liking is formed.

Although several SNPs in certain genes have been identified to be associated with the human sweet-liking trait in individuals of European ancestry^7,9,18–20^ [Table 1], whether these genes relate to sweet liking in individuals of non-European ancestry groups is unclear. It is important to note that the frequencies of these trait-increasing alleles are highly varied across individuals with different ancestry backgrounds. For example, according to the NIH dbSNP^21^, the frequencies for the trait-increasing allele *FGF21* rs838133:A are 48% in individuals of European ancestry in the U.S., 17% in individuals of Mexican ancestry in the U.S., and 1% in a Southern Han population in China. The differences in allele frequency could be one reason for the observed differences in sweet liking and added sugars intake across ancestry groups^7,22–25^. Indeed, data from the *Centers for Disease Control and Prevention* indicate that in the U.S., individuals of African ancestry have the highest average sugar intake (19 teaspoons per day), while individuals of Asian ancestry have the lowest average sugar intake (10 teaspoons per day)^26^ among ancestry groups. A list of the sweet-related genetic variants and their allele frequencies across different populations can be found in Table 2. Furthermore, the non-random associations of alleles at different loci (i.e., linkage disequilibrium, or LD) also differ by ancestry groups. For example, according to the NIH LDlink tool^27^, the associations between *FGF21* SNPs rs838133 and rs838145 are r^2^ = 0.70 in individuals of European ancestry in the U.S., r^2^ = 0.14 in individuals of African ancestry in the U.S., and r^2^ = 0.02 in Africans in Gambia, suggesting the accumulative effect of two SNPs could be different across ancestry groups. The underrepresentation of African and East Asian ancestry groups in genetics research^28^ hinders the progress in understanding how genetics may influence sweet liking in these groups. Therefore, our current study focuses on understanding the differences in the relationship between sweet-related alleles and the sweet-liking phenotype in these two groups.

Environmental exposure to sweet taste may also contribute to individual differences in sweet liking^10,12,16,17^. To investigate this, some researchers have used added sugars intake as an indicator of sweetness exposure^12^. One report assessing sugar-sweetened beverages (SSB) intake in adults in 187 countries using data from the *Global Dietary Database* showed that SSB intake in Asian countries has been lower than in Western high-income countries since 1990, whereas SSB intake in sub-Saharan Africa was lower but later increased to a similar level as Western high-income countries in 2015^29^. Theoretically, individuals who were born in countries with a lower overall added sugars intake may be less likely to be exposed to sweet foods and beverages compared to individuals who were born in countries with a higher added sugars intake. Based on the data from the *Global Dietary Database*, we can assume that most U.S.-born individuals had a greater level of sweet taste exposure during early childhood than most individuals who immigrated to the U.S. from non-Western countries. However, few studies have documented dietary changes after migrating to the U.S.^30–35^, or how these changes may affect taste liking^36^. Immigrants may retain their sweet liking developed in their home countries, as has been observed with other dietary habits^37–39^. Therefore, comparing immigrants vs. U.S.-born individuals of similar ancestry backgrounds may help inform whether environmental differences during early childhood affect sweet-liking status.

Overall, we aim to understand the genetic and environmental influences on individual differences in sweet liking by examining associations between sweet-related genetic variants and sweet liking. In particular, we will test these relationships in individuals of two ancestry groups of people in the U.S. who are underrepresented in genetic research, the African and East Asian ancestry groups. Given that most taste and genetics studies were conducted on individuals of European ancestry^7^, we will investigate whether the effects of these sweet-related genetic variants on sweet liking in other populations remain the same. We also aim to determine the differences in sweet liking between U.S.-born individuals and immigrants of the same ancestral background because their exposures to added sugars and sweet taste during early childhood may be different. Our overall goal is to enrich and expand the understanding of how genetics and environment affect sweet liking and diet in underrepresented populations living in the U.S.

## Methods and design

### Study objectives

#### Objective 1

Determine the allele frequencies and effects of sweet-related SNPs on sweet liking in two underrepresented populations – the African and the East Asian ancestry groups – living in the U.S.

We hypothesize that the frequencies of the sweet-taste-increasing alleles will be higher among individuals of African ancestry, and the associations between the sweet-related SNPs and sweet liking will be stronger compared to individuals of East Asian ancestry. We will compute ancestry-specific sweet-liking polygenic scores based on the effect sizes of the sweet-related SNPs on the sweet-liking phenotype calculated from each ancestry group. We will test the predictive validity of the polygenic scores using data from the UK Biobank participants of African and East Asian ancestries. We hypothesize that the polygenic scores will be positively associated with sweet liking and added sugars intake in the UK Biobank.

#### Objective 2

Determine the differences in sweet liking between individuals who are U.S.-born vs. immigrants within each of the African and East Asian ancestry groups living in the U.S.

We hypothesize that immigrants will have a lower degree of sweet liking compared to their U.S.-born counterparts within the same ancestry group after adjusting for individual sweet foods and beverages intake.

### Study design

**Figure 1:**
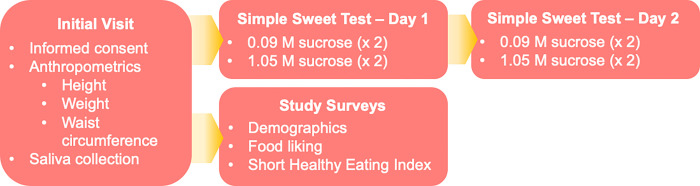
Study design flowchart.

We enroll individuals of African and East Asian ancestry groups living in the U.S. to participate in this study (see the recruitment and screening section, below). Participants attend one in-person session and two at-home sessions. During the in-person session, we take anthropometric measurements and a saliva sample for DNA extraction. Participants are sent home with two sweet taste test kits, known as the *Simple Sweet Test* kits, for their sweet liking assessment. The participants are also asked to complete a series of study questionnaires at home. These questionnaires include a validated *Food Liking Survey*^40–43^ to assess sweet foods and SSB liking, *the short Healthy Eating Index* (sHEI) *questionnaire*^44^ for assessing dietary added sugars intake and diet quality, and other dietary behavior questionnaires administered online using Qualtrics^™^. The study outcomes section below describes these procedures in detail. See Fig. 1 for a graphical representation of the study design.

Once all the samples are collected, the participants’ DNA will be extracted and sent to the *Genetic Resources Core Facility* at *Johns Hopkins University of Medicine* to be genotyped using the *Global Diversity Array*. We will assess the allele frequencies for 46 SNPs associated with sweet taste sensitivity, sweet liking/preference, and sugar/carbohydrate intake identified through GWAS and other genotype-phenotype association studies [see Table 2] and compare the difference between ancestry groups. We will estimate the effect of each SNP on sweet liking using multiple linear regression models for each ancestry group. We will use ancestry-specific SNP effects, generated in our dataset, to compute polygenic scores in the UK Biobank dataset to test its predictive validity.

### Sample size estimation

Studies by Keskitalo et al.^9^ and Knaapila et al.^13^ estimated the heritability of sweet liking response (measured by liking ratings for sweet solutions) to be 40.9% in a Finnish cohort and 27% in a U.S. cohort, respectively. To be conservative, we assumed the correlation between genetic risk and sweet liking to be r = 0.20, which yielded an effect size slope H1 of 0.06 according to analyses in G*Power^45,46^. With a type I error rate of α = 0.05 and a type II error rate of β = 0.20 (80% power), we propose to recruit 191 participants from each of the two ancestry groups, which yields a total of 191 × 2 = 382 participants for a balanced design. Accounting for a modest 10% drop-out rate, we will aim to recruit a total of 426 participants.

### Study participants and eligibility criteria

We aim to recruit 426 unrelated individuals of African and East Asian ancestry over the age of 18 with normative taste functions of all body mass index statuses. We exclude individuals who were pregnant or planning to become pregnant, those with a history of allergies to sugar, those without normal senses of smell and taste, and those without access to the internet or a device that allows them to access the internet.

To compare potential differences in sweet liking between U.S.-born individuals and those who are immigrants, it is important to code immigration status based on age at immigration. We define immigrants as those who immigrated to the U.S. after age four — a cutoff based on the completion of the complementary feeding period^47^ when food habit patterns become stable^48^. However, the age at which sweet liking becomes resistant to change is somewhat unclear. To clarify whether our immigrant distinction is sensitive to the age cutoff used, we will repeat the comparison defining immigrants as those who immigrated after age 12 (the end of middle childhood^49^) and age 19 (the end of adolescence^50^).

### Recruitment and screening

We recruit men, women, and nonbinary individuals. We anticipate a 70% female to 30% male ratio because of the sex differences in willingness to participate in research^51^. Brooklyn, NY is a particularly appropriate place for this study because of the diversity (13.6% Asian, 26.7% Black, 35.4% Caucasian, 18.9% Hispanic, and 5.4% of other)^52^ and the large number of immigrants residing in Brooklyn (approximately 38% foreign-born individuals). For individuals who were born outside of the U.S., we exclude those who moved to the U.S. less than four years of age.

We are aware that recruiting participants from racial and ethnic minority backgrounds for biomedical and clinical studies can be a challenging task. This is because of various barriers such as mistrust of the medical community, lack of transportation, location of the study center, scheduling issues, low health literacy, and stringent exclusion criteria, all of which make it difficult to recruit from this population^53–55^. To overcome these challenges, we have intentionally designed our recruitment protocol to minimize these barriers and ensure we can adequately recruit from this population^56^. The study center, Brooklyn College, is conveniently located with reliable public transportation to facilitate easy access for participants. There are no strict exclusion criteria that typically limit the eligibility of racial and ethnic minority participants based on chronic medical conditions (e.g., cardiovascular disease, Diabetes, obesity, etc.)^53,55^. Various recruitment methods will be utilized to increase the reach of study materials^57,58^. We first disseminate our recruitment materials near the Brooklyn College campus and surrounding communities, and via social media (e.g., Facebook, Instagram, TikTok) to increase the visibility of the study. We also reach our participants through professional and personal networking to establish trust and build relationships with surrounding communities^53,57,58^. Previous participants are also encouraged to invite their eligible friends and colleagues to participate in the study. Our research team is flexible and available to adapt to participants’ schedules and be transparent about the study.

### Study outcomes

#### Sweet taste liking assessment – the *Simple Sweet Test*

In the *Simple Sweet Test* for sweet taste liking assessment, participants taste sucrose solutions at varying concentrations. Solutions are prepared using food-grade sucrose (Fisher Chemical, crystalline/NF, catalog # S3–500) dissolved in distilled water. Participants first practice using the 100-point visual analog scale (VAS) by rating liking for remembered/imagined sensations. After the brief training, participants taste, using a sip-and-spit protocol, and rate their liking for two sucrose solutions (0.09 M and 1.05 M) on the 100-point VAS. Participants are instructed to rinse their mouths at least two times before tasting each sample, with a timer set for a 1-minute break between samples. Each concentration is tasted twice within the session, and the entire session is repeated on a separate day. All instructions are prompted by RedJade (RedJade Sensory Solution LLC, Martinez, CA), a SOC 2 Type II certified online software designed to collect sensory data, and is easy to follow.

The *Simple Sweet Tests* are conducted at the participant’s home. To ensure the freshness of the sucrose solutions, all solutions are prepared and used by participants within one week. We label all test solutions with a one-week expiration date and instruct our participants to not consume any samples past this date. We also instruct our participants to store the sucrose solutions in their refrigerators and only take the samples out of the refrigerator one hour before they are ready to taste the sample to allow the samples to reach room temperature.

The two sucrose concentrations from the *Simple Sweet Test* were chosen based on data from a preliminary study. In the preliminary study, we first assessed individual sweet preference using the *Monell Forced-Choice Paired Comparison Preference Tracking* test from the *NIH Toolbox for Assessment of Neurological and Behavioral Function*^59,60^, during which participants tasted five concentrations of sucrose (0.09, 0.18, 0.35, 0.70, 1.05 M) dissolved in water and were asked to choose their preferred sample in a forced-choice paradigm. On a separate occasion, we asked the same group of participants to rate the same 5 sucrose concentrations using a 100-point VAS in duplicates. The 100-point visual analog scale is the scaling method used in the *Simple Sweet Test* because it is intuitive and easy to use, a key advantage in self-administered taste tests, among other psychometric strengths^61,62^. We found that liking ratings from 2 concentrations (0.09 M and 1.05 M) explained 76% of the variance of the most preferred concentration identified using the *Monell Forced-Choice Paired Comparison Preference Tracking* test^63^. This demonstrated that using only the liking ratings of 2 sucrose concentrations, 0.09 M and 1.05 M, could predict the preferred concentration with reasonable accuracy^63^.

**Figure 2:**
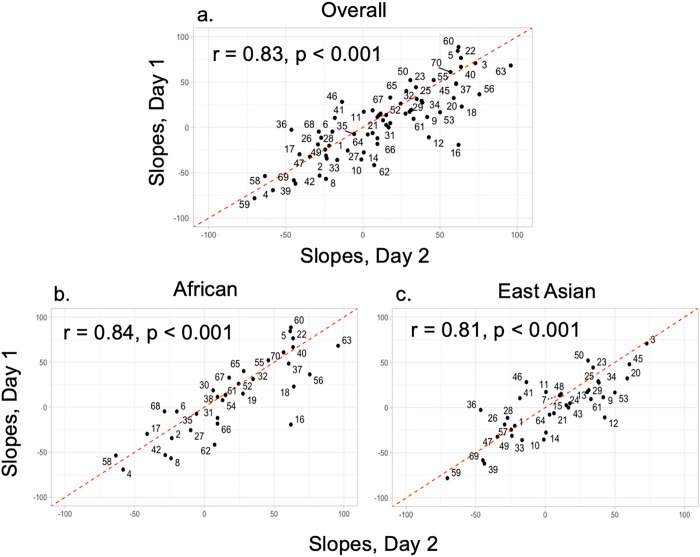
Test-retest reliability of the *Simple Sweet Test*between 2 different test days. On the top is the test-retest reliability of all participants. On the bottom left is the test-retest reliability of the African ancestry group. On the bottom right is the test-retest reliability of the East Asian ancestry group. The number on the plot represents participant IDs.

**Figure 3:**
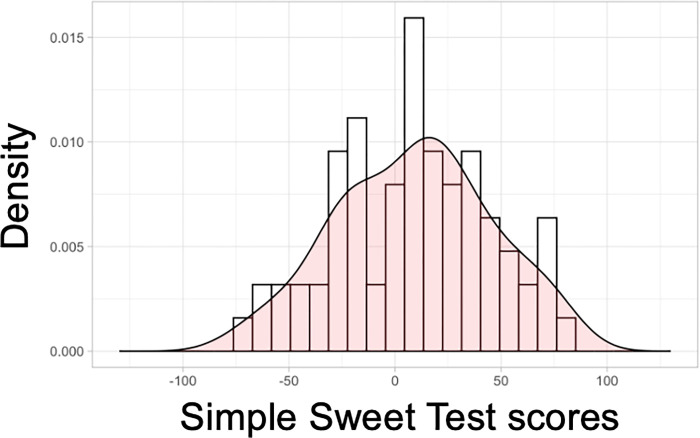
Distribution of the sweet liking phenotype assessed using the *Simple Sweet Test*.

The congruency between taste-liking tests in a traditional laboratory setting compared to taste tests conducted at home has been explored in a previous study^64^. The correlation between sweet-liking ratings obtained in the laboratory vs. at home was high, with a correlation coefficient of r = 0.86. Furthermore, taste tests for liking conducted at home provide better ecological validity, as people typically do not consume sweet foods or beverages in a laboratory setting. Another advantage of the *Simple Sweet Test* is that it only takes approximately 10 minutes to complete, thus lowering participants’ burden and reducing the risk of sensory fatigue. The entire test is repeated on a separate day. The two test sessions are replicated sessions to ascertain test-retest reliability using Pearson’s correlation coefficient. An individual sweet liking score is calculated using the slopes of the degree of liking for the 0.09 M and 1.05 M solutions. Our pilot data indicates that the *Simple Sweet Test* has high test-retest reliability in both the African and the East Asian groups (Pearson’s correlation r = 0.81 to 0.84, p < 0.001) [Figure 2]. The *Simple Sweet Test* can also capture a wide range of sweet-liking responses [Figure 3]. Overall, the at-home *Simple Sweet Test* is a reliable method to collect high-quality data.

#### Sweet food and beverage liking assessment – the *Food Liking Survey*

Participants complete a validated *Food Liking Survey* developed by Duffy et al.^40–43^ A version of this survey is administered in the UK Biobank^65^. The modified version of the *Food Liking Survey* in our study contains 66 items (including 6 non-food items), divided into food groups based on their sensory and nutritional qualities, on a 100-point scale anchored with the extremes “hate it” and “love it” from this survey^40–43^. Out of the 60 foods and beverages, 5 items (ice cream, cookies/cakes/pastries, sweet confectionaries/candies, jelly/jam/syrup, and chocolate) are grouped into a “sweets” category and 4 items (soda/soft drinks, sweetened coffee drinks, energy drinks, and juice/sweet tea/smoothies) are grouped into a “sugar-sweetened beverages” category. Other food groups include vegetables, fruits, salty/fat, high-fat protein, alcoholic drinks, whole grains/fiber, refined grains/carbs, healthy fat, unhealthy fat, lean protein, and spicy. A list of the food groups and food items within each group can be found in Table 3.

The *Food Liking Survey* has been validated in different ancestral groups^66^. A sweet food and beverage liking score will be calculated from survey results, an approach previously used to quantify the degree of liking for sweetness^67^. Cronbach’s alpha will be used to assess the internal consistency of the food items within each group.

#### Sweet exposure assessment – the *short Health Eating Index questionnaire*

Habitual added sugars intake will be assessed as an indicator of sweetness exposure using the *short Healthy Eating Index*, or sHEI. The *sHEI questionnaire* is a rapid and cost-efficient method to estimate food intake and is validated for estimating overall dietary quality^44^. The 22-item sHEI has previously been shown to be a reliable proxy for added sugars and sugar-sweetened beverage intake frequencies^44^. The frequencies of sugar-sweetened beverages, sugary foods, and added sugars consumption are used to calculate an estimated daily added sugars intake value ranging from 0 to 1456 kcals per day. Individual added sugars intake kcals per day values were divided by four to obtain the daily added sugars intake per day in grams.

#### Saliva collection and genotyping

Participants are asked to expectorate into a DNA collection kit, which contains a small tube with a fill line (~ 2 mL) for saliva samples (Oragene.Discovery, Ottawa, Ontario, Canada). We instruct the participants to not eat or drink anything within 1 hour of providing the saliva sample. Saliva samples are stored at City University of New York, Brooklyn College until analysis at −20°C. The samples will be shipped to the *Genetic Resources Core Facility* at *Johns Hopkins University of Medicine* and analyzed using the *Global Diversity Array*, the gold standard in human genotyping and used by the *NIH All-of-Us* program.

Aside from genotyping for the sweet-related alleles listed in Table 1, we will also use the participants’ genetic information to infer their ancestry groups based on their genetic distance scores to several reference populations (e.g., 1000 genome) using the GRAF-pop program^68^. The inferred ancestries will be harmonized with self-identified race/ethnicity. We will be able to confirm participants’ self-identified ancestry groups using this method in the study. Based on preliminary analyses of related research, we expect self-reported race data to fail to match ancestry groups based on GRAPH-pop results for approximately 2% of participants. Those participants will be excluded from the main analyses. This percentage is incorporated into the drop-out rate described above.

#### Anthropometrics measures

We will measure participants’ height and weight. Height and weight measures are taken with light clothing without footwear. Height and weight are measured three times to the nearest 0.25 inches using a stadiometer with a balance beam (Newell Brands Inc., Atlanta, GA).

#### UK Biobank dataset

The UK Biobank is a population-based prospective study consisting of over 500,000 participants (aged 40–69 years; 54.4% women; 5% of those invited) recruited across 22 assessment centers in the United Kingdom between 2006 and 2010^69^. Participants responded to questionnaires to provide information on health and lifestyle in a baseline survey, took part in clinical assessments, and provided biological samples for biomarker and genetic assays. Participants also completed a food frequency questionnaire while visiting the assessment centers and a follow-up online 24-hour dietary recall, from the latter free sugar intake was estimated from the overall diet. Food-liking traits were collected through a version of the *Food Liking Survey* with 152 items, including 139 food and drink items plus additional non-food items that captured liking for health-related behaviors such as physical activity. Participants rated their liking on a 9-point hedonic scale, with 1 corresponding to “Extremely dislike” and 9 to “Extremely like”. The questionnaire was administered in 2019 to all UK Biobank participants who had agreed to be recontacted by the study.

#### Statistical analyses plan

Transforms on outcome variables will be considered prior to analyses if assumptions for the linear regression model are violated. We will analyze data missingness for non-random patterns based on associations with other participant data, including age, ancestry, sweet liking, and anthropomorphic measures. Missingness found to be associated with other factors will be incorporated into our interpretation of results. Participants’ data will be analyzed separately according to their ancestry groups.

For *Objective 1*, we will compare the allele frequencies of the sweet-related SNPs for the African and East Asian ancestry groups living in the U.S. separately. The differences in allele frequencies will be tested using chi-square tests. Multivariate linear regression analyses will be conducted to determine the association and the beta effect size between the sweet-related SNPs from Table 1 and the phenotype (i.e., sweet liking). The influence of age and sex on sweet liking will be assessed, and if they are significantly associated with sweet liking they will be entered into the model as covariates. The calculations will be conducted separately for individuals of African and East Asian ancestries as the results may differ between ancestry groups.

From there, we will use the SNPs associated with the sweet liking phenotype from Table 1 and the beta effect sizes of the SNPs from to create polygenic scores for the African and East Asian ancestry groups. The polygenic scores will be weighted based on their effect sizes for each ancestry group. For SNPs in ancestry-specific LD of r^2^ > = 0.8, only one SNP will be included in the calculation of polygenic scores. For SNPs in ancestry-specific LD of r^2^ < 0.8 and r^2^ > 0.5, we will fit all SNPs in one multivariate linear regression model and use the conditional beta effect sizes to calculate polygenic scores. We will also calculate unweighted polygenic scores by summing the number of sweet-liking-increasing alleles.

We will use the same scoring method developed using the U.S. cohort on a separate data set (i.e., the *UK Biobank*) to predict sweet food/beverage liking and sugar intake to determine the predictive validity of the polygenic scores. We will apply this scoring method for the UK Biobank participants of African (n = 10149) and East Asian (n = 8219) ancestries. Sweet food/beverage liking information collected using the *Food Liking Survey* in the UK Biobank will be averaged into a sweet food and beverage liking score. We will then determine the associations between the individual polygenic scores and sweet food and beverage liking scores for both ancestry groups within the UK Biobank data set. With a sample size of 8219, we have 80% power (alpha = 0.05) to detect an effect if the polygenic score accounts for > 0.12% of the variance in sweet liking scores.

For *Objective 2*, multivariate linear regression analyses will be conducted to determine the association between immigration status and sweet liking within each ancestry group. We will compare the mean sweet-liking scores between immigrants and non-immigrants using a t-test. We will first regress sweet-liking scores on the covariates of age and sex and then take the residuals for comparison. Then we will further regress sweet liking scores on age, sex, and added sugar intake and take residuals for comparison to investigate the influence of sweet taste exposure on sweet liking.

## Discussion

This study aims to assess the effects of genetics and environment on sweet liking and added sugars intake by studying individuals from two underrepresented ancestry groups in the U.S., the African and the East Asian ancestry groups. It is important to point out that the goal of this study is to understand how individuals differ from one another within these groups, but not to make general assumptions about the groups. While studies that investigated the average differences in sweet liking across ancestry groups provide a starting point for untangling these relationships^39,70^, it is also important to understand how genetic makeup and previous experiences with foods affect sweet liking within underrepresented communities on an individual level, similar to the efforts that have been made to understand individual differences within European communities^7,13,19^. For example, in a study that explored the relationship between variations in *TAS2R38*, a gene that is associated with bitter sensitivity, and sweet preference within children and mothers of African and European ancestries living in the U.S.^71^, it was found that *TAS2R38* is associated with sweet preference in children but not in their mothers^71^, with individuals of African descent preferred a higher sweetness level compared to individuals of European descent. It was concluded from this study that cultural forces and experience may contribute to a significant proportion of variance in sweet liking thus overriding the genetic effects observed in children^71^. In the current study, we expand upon these findings^71^ to account for the effects of multiple genes that have been associated with sweetness perception, intake, and liking in two populations that are underrepresented in science. Our goal is to provide a more comprehensive picture of how genetics may influence sweet liking and added sugars intake using a polygenic approach.

There is a lack of large-scale genomic data sets directly assessing sweet liking as a phenotype. A few studies used sugar intake and intensity perception as proxy measures for the sweet liking phenotype^7,20^. Although liking is associated with consumption^1–3^, it does not always predict consumption^1,72,73^. Therefore, it is possible that added sugars intake may not be a reliable or appropriate assessment of individual liking for sweet tastes. With that in mind, there is a need to collect the sweet-liking phenotype using a reliable and accessible method. A key strength of the current study is that we use the *Simple Sweet Test*, a method developed to allow for the collection of large-scale sweet-liking phenotype data. With the *Simple Sweet Test*, participants can take the test in the comfort of their own homes, an environment where sweet foods and beverages are more typically consumed compared to the traditional laboratory setting. Deploying more tests that can be conducted at home, such as the *Simple Sweet Test*, is critical to increasing the accessibility of taste and genetic research to more diverse populations in the future.

We acknowledge that the acculturation of immigrants could be a potential confounder for determining the environmental effects on sweet liking because it may affect individual added sugars intake. Therefore, the immigrant group only includes individuals who moved to the U.S. aged 4 years or older, after the complementary feeding period when toddlers were first introduced to solid foods^47,74^. Furthermore, we will test whether individualized added sugars intake is a confounder and add this as a covariate in our statistical models if appropriate. We also acknowledge that immigrants are from many ethnically diverse backgrounds (e.g., East Asians from Japan vs. the Hmong people from Laos, Black Jamaican vs. Black Africans). This study is not powered to analyze the differences across immigrant groups, but instead, we focus on whether differences exist between U.S.-born individuals and immigrants as a first step to understanding this complex topic. We also acknowledge that non-nutritive sweetener exposure may interfere with study outcomes. We aim to focus on the effects of sugars in this study, but we will attempt to quantify the effects of non-nutritive sweeteners in future studies.

## Conclusion

There is a need to consider individual taste liking when providing culturally appropriate diet recommendations. In this study, we aim to understand what drives sweet liking and added sugars intake in underrepresented populations. Data from this study will be a step toward devising personalized nutrition guidance based on individual taste preferences. A few studies are currently underway to test whether sweet liking/preference can be shifted (ClinicalTrials.gov ID: NCT04079855, NCT04497974), similar to the shift in saltiness liking after a low-salt diet^75^. Our results will aid in developing tailored approaches to modify sweet liking based on individualized genetic and environmental factors. The overall goal is to expand our knowledge of taste preference genotype-phenotype associations and their interactions with environmental factors beyond the European ancestry group to inform research and interventions for all populations.

### Study status

The study recruitment began in July 2023 and is currently ongoing. The projected end date of this project is June 2027.

## Figures and Tables

**Table 1:** Sweet-related single nucleotide polymorphisms and their corresponding phenotypes

Chr	Gene	SNP	EA	Study Population	Phenotypes
1	*TAS1R2*	rs12033832	A	n = 696 (unknown ancestry); Toronto Nutrigenomics and Health study	Higher sucrose sensitivity (lower detection threshold) and lower sugar intake (grams per day) among those with BMI ≥ 25; opposite associations among those with BMI < 25^76^
				n = 30 (unknown ancestry); an Australian cohort	Higher percentage energy intake from carbohydrates in an ad libitum meal session ≤ 40 min^77^
				n = 144; 92 Europeans, 37 Asians, 15 Africans	No association with sucrose sensitivity^78^
1	*TAS1R2*	rs3935570	T	n = 696 (unknown ancestry); Toronto Nutrigenomics and Health study	Higher sucrose sensitivity (lower detection threshold) among those with BMI ≥ 25; no association with sugar intake (grams per day) regardless of BMI^76^
1	*TAS1R2*	rs35874116	A	n = 1037 (unknown ancestry); 482 European, 362 East Asians, 114 South Asians, 79 others; Toronto Nutrigenomics and Health study	Higher intake of carbohydrates (grams per day) and sugar (grams per day) among those with BMI ≥ 25^79^
				n = 100 (unknown ancestry); individuals with diabetes from the Canadian Trial of Carbohydrate in Diabetes multicenter intervention study	Higher intake of sugar (grams per day)^80^
				n = 312 children (43.2% white); a Brazilian cohort	Higher sugar intake (kilocalories per day) at age 3.9 y; no association at ages 1.1 and 7.7 y^81^
				n = 30 (unknown ancestry); an Australian cohort	Higher intake of sweets (grams) in an ad libitum meal session ≤ 40 min^77^
				n = 441 (Mestizos); a West Mexican cohort	Lower intake of carbohydrates (grams per day) and percentage energy intake from carbohydrate^82^
				n = 47 children (87.5% Caucasian); the Guelph Family Health Study	Higher percentage energy intake from snacks^83^
				n = 696 (unknown ancestry); Toronto Nutrigenomics and Health study	No association between sucrose sensitivity and sugar intake (grams per day)^76^
				n = 144; 92 Europeans, 37 Asians, 15 Africans	No association with sucrose sensitivity^78^
1	*TAS1R2*	rs7534618	A	n = 65 adults (85% Caucasian) and 60 children (81% Caucasian); the Guelph Family Health Study	Association with a preference for sucrose solution among children^84^
1	*TAS1R2*	rs28652778	T	n = 127 adults (all Europeans); a Swedish cohort	Higher preference for sweetness^85^
1	*TAS1R3*	rs307355	C	n = 144 (92 Europeans, 37 Asians, 15 Africans)	Higher sucrose sensitivity^78^
				n = 30 (unknown ancestry); an Australian cohort	No association with percentage energy intake from carbohydrates or intake of sweets (grams) in an ad libitum meal session ≤ 40 min^77^
1	*TAS1R3*	rs35744813	C	n = 144 (92 Europeans, 37 Asians, 15 Africans)	Higher sucrose sensitivity^78^
				n = 76 mothers (32.9% white, 52.6% black, 5.3% Hispanic/Latino/Latina, 1.3% Asian, 7.9% others) and 101 children (31.7% white, 42.6% black, 8.9% Hispanic/Latino/Latina, 2% Asian, 14.9% others); a U.S. cohort	Preference for a sucrose solution of a lower concentration among mothers; no association in children^86^
				n = 235 children (46 whites, 136 blacks, 2 Asians, 51 others); a U.S. cohort	No association between sucrose detection threshold and percentage energy intake from added sugar^87^
				n = 312 children (43.2% white); a Brazilian cohort	No association with sugar intake (kilocalories per day) ^81^
				n = 30 (unknown ancestry); an Australian cohort	No association with percentage energy intake from carbohydrates or intake of sweets (grams) in an ad libitum meal session ≤ 40 min^77^
2	*AC007100.1*	rs12713415	C	n = 235,391 (all Europeans) UK Biobank + replication cohorts	Lower sugar intake^88^
2	*AC007100.1*	rs10206338	A	n = 235,391 (all Europeans) UK Biobank + replication cohorts	Lower sugar intake^88^
3	*SLC2A2*	rs5400	T	n = 1037 (482 European, 362 East Asians, 114 South Asians, 79 others); Toronto Nutrigenomics and Health study	Higher intake of carbohydrates (grams per day) and sucrose (grams per day) ^79^
				n = 100 (unknown ancestry); individual with diabetes from the Canadian Trial of Carbohydrate in Diabetes multicenter intervention study	Higher sugar intake (grams per day) ^80^
				n = 22,799 (unknown ancestry); a Swedish cohort	Lower carbohydrate intake^89^
3	*RARB*	rs10510554	T	n = 268,922 (all Europeans); UK Biobank + replication cohorts	Higher carbohydrate intake^88^
3	*RARB*	rs7619139	A	n = 235,391; (all Europeans); UK Biobank + replication cohorts	Lower sugar intake^88^
3	*CADM2*	rs10433500	A	n = 268,922; (all Europeans); UK Biobank + replication cohorts	Higher carbohydrate intake^88^
6	*SNORD66*	rs13202107	A	n = 235,391; (all Europeans); UK Biobank + replication cohorts	Lower sugar intake^88^
7	*GNAT3*	rs7792845	T	n = 160 (103 Caucasians, 41 Asians, 16 Africans); a US cohort	Higher sucrose sensitivity^90^
7	*GNAT3*	rs940541	T	n = 160 (103 Caucasians, 41 Asians, 16 Africans); a US cohort	Higher sucrose sensitivity^90^
7	*GNAT3*	rs1107660	T	n = 160 (103 Caucasians, 41 Asians, 16 Africans); a US cohort	Higher sucrose sensitivity^90^
				n = 22,799 (unknown ancestry); a Swedish cohort	Lower carbohydrate intake^89^
7	*GNAT3*	rs1107657	T	n = 160 (103 Caucasians, 41 Asians, 16 Africans); a US cohort	Higher sucrose sensitivity^90^
7	*GNAT3*	rs1524600	C	n = 160 (103 Caucasians, 41 Asians, 16 Africans); a US cohort	Higher sucrose sensitivity^90^
7	*GNAT3*	rs6467217	T	n = 160 (103 Caucasians, 41 Asians, 16 Africans); a US cohort	Higher sucrose sensitivity^90^
7	*GNAT3*	rs6970109	C	n = 160 (103 Caucasians, 41 Asians, 16 Africans); a US cohort	Higher sucrose sensitivity^90^
7	*GNAT3*	rs6975345	T	n = 160 (103 Caucasians, 41 Asians, 16 Africans); a US cohort	Higher sucrose sensitivity^90^
7	*GNAT3*	rs10242727	A	n = 160 (103 Caucasians, 41 Asians, 16 Africans); a US cohort	Higher sucrose sensitivity^90^
7	*GNAT3*	rs6467192	G	n = 160 (103 Caucasians, 41 Asians, 16 Africans); a US cohort	Higher sucrose sensitivity^90^
7	*GNAT3*	rs6961082	C	n = 160 (103 Caucasians, 41 Asians, 16 Africans); a US cohort	Higher sucrose sensitivity^90^
8	*AC022784.6*	rs7012637	A	n = 268,922 (all Europeans); UK Biobank + replication cohorts	Higher carbohydrate intake^88^
8	*AC022784.6*	rs7012814	A	n = 235,391 (all Europeans); UK Biobank + replication cohorts	Higher sugar intake^88^
8	*AC073284.4*	rs7424551	G	n = 174,424 (all Europeans); UK Biobank	Higher intake of sugars^7^
16	*FTO*	rs11642841	C	n = 174,424 (all Europeans); UK Biobank	Higher intake of sugars but not sweets; Lower BMI^7^
16	*FTO*	rs55872725	T	n = 422,300 (all Europeans); UK Biobank	Higher intake of sugar-sweetened beverages^14^
16	*FTO*	rs9939609	A	n = 22,799 (unknown ancestry); a Swedish cohort	Higher intake of sugar-sweetened beverages^89^
16	*FTO*	rs9972653	T	n = 235,391 (all Europeans) UK Biobank + replication cohorts	Lower sugar intake^88^
16	*FTO*	rs7190396	T	n = 268,922 (all Europeans); UK Biobank + replication cohorts	Higher carbohydrate intake^88^
16	*ZFHX3*	rs1104608	C	n = 268,922 (all Europeans); UK Biobank + replication cohorts	Higher carbohydrate intake^88^
17	*SLC2A4*	rs2654185	A	n = 127 adults (all Europeans); a Swedish cohort	Higher sweet taste threshold; less intake of sweet foods^85^
17	*SLC2A4*	rs5415	T	n = 127 adults (all Europeans); a Swedish cohort	Higher sweet taste threshold^85^
17	*SLC2A4*	rs5418	G	n = 127 adults (all Europeans); a Swedish cohort	Less intake of sweet foods^85^
17	*ARL17B*	rs36123991	T	n = 268,922 (all Europeans); UK Biobank + replication cohorts	Higher carbohydrate intake^88^
18	*AP005230.1*	rs8097672	A	n = 235,391 (all Europeans); UK Biobank + replication cohorts	Higher sugar intake^88^
				n = 268,922 (all Europeans); UK Biobank + replication cohorts	Higher carbohydrate intake^88^
18	*L3MBTL4*	rs341228	T	n = 235,391 (all Europeans); UK Biobank + replication cohorts	Higher sugar intake^88^
19	*FGF21*	rs838133	A	n = 33,533 (all Europeans); the DietGen Consortium	Lower percentage energy intake from carbohydrate^91^
				n = 6515 (all Europeans); a Danish cohort	Higher weekly intake of sweet snacks and candies^20^
				n = 176,989 (all Europeans); UK Biobank	Higher percentage energy intake from carbohydrate^92^
				n = 22,799 (unknown ancestry); a Swedish cohort	Higher intake of total sugars and added sugars^89^
19	*FGF21*	rs838145	G	n = 38,360 (all Europeans); the CHARGE Consortium	Higher percentage energy intake from carbohydrate^93^
				n = 22,799 (unknown ancestry); a Swedish cohort	Higher intake of total sugars and added sugars^89^
19	*FGF21*	rs8103840	C	n = 22,799 (unknown ancestry); a Swedish cohort	Higher intake of total sugars and added sugars^89^
19	*FGF21*	rs62132802	T	n = 235,391 (all Europeans); UK Biobank + replication cohorts	Lower sugar intake^88^
19	*APOE*	rs429358	T	n = 235,391 (all Europeans); UK Biobank + replication cohorts	Lower sugar intake^88^
				n = 268,922 (all Europeans); UK Biobank + replication cohorts	Lower carbohydrate intake^88^
19	*IZUMO1*	rs838144	T	n = 235,391 (all Europeans); UK Biobank + replication cohorts	Lower sugar intake^88^
				n = 268,922 (all Europeans); UK Biobank + replication cohorts	Lower carbohydrate intake^88^

Chr = chromosome, SNP = single nucleotide polymorphism, EA = effect allele

**Table 2:** Sweet-related single nucleotide polymorphisms and their effect allele frequencies across populations

Chr	Gene	SNP	EA	EAF Global	EAF EUR	EAF AFR	EAF ASI
1	*TAS1R2*	rs12033832	A	0.31237	0.32579	0.2378	0.418
1	*TAS1R2*	rs3935570	T	0.260578	0.266728	0.2674	0.093
1	*TAS1R2*	rs35874116	T	0.67936	0.670589	0.67866	0.8667
1	*TAS1R2*	rs7534618	T	0.65273	0.644773	0.75479	0.537
1	*TAS1R2*	rs28652778	T	0.224534	0.239208	0.10282	0.0013
1	*TAS1R3*	rs307355	C	0.8658	0.93091	0.5754	0.88
1	*TAS1R3*	rs35744813	C	0.83822	0.92972	0.4162	0.88
2	*AC007100.1*	rs12713415	C	0.73936	0.73799	0.7258	0.94
2	*AC007100.1*	rs10206338	A	0.61554	0.60633	0.6315	0.402
2	*AC073284.4*	rs7424551	G	0.36769	0.65415	0.4277	1
3	*SLC2A2*	rs5400	T	0.147852	0.136075	0.45245	0.0068
3	*RARB*	rs10510554	T	0.44018	0.41026	0.5754	0.438
3	*RARB*	rs7619139	A	0.57199	0.60782	0.4131	0.589
3	*CADM2*	rs10433500	A	0.57501	0.65197	0.1993	0.893
6	*SNORD66*	rs13202107	A	0.10787	0.13913	0.0141	0.02
7	*GNAT3*	rs7792845	T	0.38754	0.40456	0.1891	0.273
7	*GNAT3*	rs940541	T	0	0	0	0
7	*GNAT3*	rs1107660	T	0.363084	0.372784	0.1543	0.2626
7	*GNAT3*	rs1107657	T	0.33404	0.36784	0.1583	0.25
7	*GNAT3*	rs1524600	C	0.869609	0.89758	0.5224	0.817
7	*GNAT3*	rs6467217	T	0.85087	0.90767	0.5726	0.72
7	*GNAT3*	rs6970109	C	0.89597	0.902003	0.6788	0.879
7	*GNAT3*	rs6975345	T	0.861231	0.87928	0.3673	0.8686
7	*GNAT3*	rs10242727	A	0.82533	0.88659	0.3336	0.773
7	*GNAT3*	rs6467192	G	0.861056	0.879454	0.3723	0.84
7	*GNAT3*	rs6961082	C	0.940063	0.943922	0.866	0.871
8	*AC022784.6*	rs7012637	A	0.29626	0.3485	0.0241	1
8	*AC022784.6*	rs7012814	A	0.58192	0.64357	0.0249	1
16	*FTO*	rs11642841	C	0.627376	0.602175	0.9318	0.954
16	*FTO*	rs55872725	T	0.38661	0.4275	0.089	0.171
16	*FTO*	rs9939609	A	0.39857	0.41025	0.4744	0.1246
16	*FTO*	rs9972653	T	0.57919	0.62542	0.4151	0.384
16	*FTO*	rs7190396	T	0.73648	0.6986	0.94	1
16	*ZFHX3*	rs1104608	C	0.43268	0.40831	0.6034	0.28
17	*SLC2A4*	rs2654185	A	0.398241	0.367033	0.6812	0.65
17	*SLC2A4*	rs5415	T	0.284901	0.292429	0.1007	0.317
17	*SLC2A4*	rs5418	G	0.436197	0.412875	0.7041	0.6404
17	*ARL17B*	rs36123991	T	0.12469	0.17097	0.0389	0
18	*AP005230.1*	rs8097672	A	0.80889	0.85139	0.5828	1
18	*L3MBTL4*	rs341228	T	0.28523	0.33095	0.0682	0.24
19	*FGF21*	rs838133	A	0.424156	0.4431	0.3303	0.01
19	*IZUMO1*	rs838145	G	0.31663	0.39468	0.0834	0
19	*FUT1*	rs8103840	C	0.64465	0.6059	0.934	0.9
19	*FGF21*	rs62132802	T	0.30614	0.32465	0.224	0.2
19	*APOE*	rs429358	T	0.925582	0.93183	0.8712	0.974
19	*IZUMO1*	rs838144	T	0.5939	0.534	0.7406	1

Chr = chromosome, SNP = single nucleotide polymorphism, EA = effect allele, EAF = effect allele frequency, EUR = European, AFR = African, ASl = East Asian

**Table 3:** Food liking survey items and food groups

Food groups	Food items
Vegetable	Dark green leafy vegetables (e.g., spinach, kale, collard greens, arugula)
	Broccoli/cauliflower/cabbage/bok choy
	Tomatoes
	Zucchini, squash, eggplant
	Cucumbers
	Carrots (raw or cooked)
Fruit	Bananas
	Oranges, grapefruits, tangerines
	Apples/pears
	Berries
	Melon (any variety)
Salty/fat	Salty snacks (e.g., chips, crackers, popcorn)
	French fries
	Fast food
	Pickles
High-fat protein	Fried meats (e.g., Chicken, fish, pork, beef)
	Bacon/sausage
	Beef steak
	Pizza
	Fatty deli meats (e.g., pepperoni, salami, pastrami)
Alcoholic	Beer
	Liquor/distilled spirits (e.g., tequila, vodka, rum, whiskey)
	Wine
	Hard seltzers
	Mixed drinks/cocktails
Whole Grain/fiber	Whole wheat bread or tortilla
	Whole wheat cereal flakes/oatmeal/granola
	Beans/lentils
	Brown rice
Refined grain/carbohydrate	White rice
	White bread/dinner rolls/white flour tortilla
	Spaghetti/pasta/noodles
	White potatoes
	Sugary cereal (e.g., Trix, Fruit Loops)
Healthy fat	Nuts & seeds (any variety)
	Nut butter (any variety)
	Olive oil
	Avocado
Sweets	Ice cream
	Cookies/cakes/pastries
	Sweet confectionaries/candies
	Jelly/jam/syrup
	Chocolate (milk or white)
Sugar-sweetened beverages	Soda/soft drinks (e.g., Cola, Pepsi, Sprite)
	Sweetened coffee drinks (e.g., latte, cappuccino)
	Energy drinks
	Juice/sweet tea/smoothies
Unhealthy fat	Cheese (any variety)
	Whole milk
	Mayonnaise
	Butter/lard/ghee
	Vegetable oil
Lean protein	Chicken/turkey (baked, grilled, or roasted)
	Tuna fish
	Soy or alternative vegetable-based protein products
	Goat meat
	White flaky fish (e.g., cod, tilapia, haddock)
Spicy	Tabasco/hot sauce
	Chili sauce
	Mustard/horseradish
Experience	Seeing a mouse in your house
	Being late for an important date
	Watching your favorite team win
	Receiving a compliment
	Smell of garbage
	Going on vacation/holiday

## Abbreviations

GWAS: Genome-wide association studies
LD: Linkage disequilibrium
sHEI: short Healthy Eating Index
SNP Single: nucleotide polymorphism
SSB Sugar: sweetened beverages
U.S.: United States
VAS: Visual analog scale
